# POstmastectomy radioThErapy in Node-posiTive breast cancer with or without Internal mAmmary nodaL irradiation (POTENTIAL): a study protocol for a multicenter prospective phase III randomized controlled trial

**DOI:** 10.1186/s12885-021-08852-y

**Published:** 2021-11-06

**Authors:** Xu-Ran Zhao, Hui Fang, Yu Tang, Zhi-Hui Hu, Hao Jing, Lin Liang, Xue-Na Yan, Yong-Wen Song, Jing Jin, Yue-Ping Liu, Bo Chen, Yuan Tang, Shu-Nan Qi, Ning Li, Ning-Ning Lu, Kuo Men, Chen Hu, Yu-Hui Zhang, Ye-Xiong Li, Shu-Lian Wang

**Affiliations:** 1 Department of Radiation Oncology, National Cancer Center/National Clinical Research Center for Cancer/Cancer Hospital, Chinese Academy of Medical Sciences and Peking Union Medical College, Beijing, 100021 China; 2 Heart Failure Center, State Key Laboratory of Cardiovascular Disease, Fuwai Hospital, National Center for Cardiovascular Diseases, Chinese Academy of Medical Sciences and Peking Union Medical College, No. 167 Beilishi Road, Xicheng District, Beijing, 100037 China; 3 Division of Biostatistics and Bioinformatics, Sidney Kimmel Comprehensive Cancer Center, Johns Hopkins University School of Medicine, Baltimore, MD 21205-2013 USA

**Keywords:** Breast cancer, Postmastectomy radiotherapy, Internal mammary node irradiation, Survival outcome, Toxicity

## Abstract

**Background:**

Various randomized trials have demonstrated that postmastectomy radiotherapy (RT) to the chest wall and comprehensive regional nodal areas improves survival in patients with axillary node-positive breast cancer. Controversy exists as to whether the internal mammary node (IMN) region is an essential component of regional nodal irradiation. Available data on the survival benefit of IMN irradiation (IMNI) are conflicting. The patient populations enrolled in previous studies were heterogeneous and most studies were conducted before modern systemic treatment and three-dimensional (3D) radiotherapy (RT) techniques were introduced. This study aims to assess the efficacy and safety of IMNI in the context of modern systemic treatment and computed tomography (CT)-based RT planning techniques.

**Methods:**

POTENTIAL is a prospective, multicenter, open-label, parallel, phase III, randomized controlled trial investigating whether IMNI improves disease-free survival (DFS) in high-risk breast cancer with positive axillary nodes (pN+) after mastectomy. A total of 1800 patients will be randomly assigned in a 1:1 ratio to receive IMNI or not. All patients are required to receive ≥ six cycles of anthracycline and/or taxane-based chemotherapy. Randomization will be stratified by institution, tumor location (medial/central vs. other quadrants), the number of positive axillary nodes (1–3 vs. 4–9 vs. ≥10), and neoadjuvant chemotherapy (yes vs. no). Treatment will be delivered with CT-based 3D RT techniques, including 3D conformal RT, intensity-modulated RT, or volumetric modulated arc therapy. The prescribed dose is 50 Gy in 25 fractions or 43.5 Gy in 15 fractions. Tiered RT quality assurance is required. After RT, patients will be followed up at regular intervals. Oncological and toxilogical outcomes, especially cardiac toxicities, will be assessed.

**Discussion:**

This trial design is intended to overcome the limitations of previous prospective studies by recruiting patients with pN+ breast cancer, using DFS as the primary endpoint, and prospectively assessing cardiac toxicities and requiring RT quality assurance. The results of this study will provide high-level evidence for elective IMNI in patients with breast cancer after mastectomy.

**Trial registration:**

ClinicalTrails.gov, NCT04320979. Registered 25 Match 2020, https://clinicaltrials.gov/ct2/show/NCT04320979

**Supplementary Information:**

The online version contains supplementary material available at 10.1186/s12885-021-08852-y.

## Background

The survival benefits of postmastectomy radiation therapy (PMRT) in axillary node-positive breast cancer have been confirmed by prospective randomized trials and meta-analyses [1–4]. In all these studies, the treatment volume included the chest wall plus comprehensive regional nodal areas. The internal mammary node (IMN) chain is one of the major pathways of lymphatic drainage in breast cancer. Historical surgical series have demonstrated that the incidence of IMN metastasis was 15.5% in patients after extended radical mastectomy [5] and IMN metastasis was more often observed in patients with positive axillary nodes or medial tumors compared with those without [6]. Although the clinical recurrence rate in an untreated IMN region is rare, 1.5% [7], the subclinical disease harbored in these nodes might subsequently metastasize to distant areas without manifesting in the IMN region.

Early randomized trials, conducted in the era before the availability of systemic therapy, showed that IMN dissection did not improve overall survival (OS) [8, 9]. As a result, IMN dissection is abandoned. However, patients with a positive IMN had poorer OS than those with a negative IMN [9]. There is ongoing controversy regarding whether the IMN should be included in the radiation treatment volume, largely because of the unconfirmed survival benefit and the concerns for added risk of cardiac toxicity from IMN irradiation (IMNI). Two large prospective studies evaluating the specific role of IMNI showed conflicting results. A French randomized trial showed no overall survival benefit associated with IMNI in patients with node-positive or high-risk node-negative breast cancer after mastectomy [10]. While a multicenter, population-based, prospective cohort study launched by the Danish Breast Cancer Cooperative Group (DBCG) demonstrated that IMNI increased OS significantly in patients with node-positive breast cancer [11]. It should be noted that the patient populations enrolled in these two studies were different and both studies were conducted before modern systemic treatment was introduced. The OS benefit from IMNI is highly dependent on the efficacy of systemic therapy. It is hypothesized that patients will benefit most from IMNI if systemic therapy is effective to control the distant disease, but not sufficient to sterilize the subclinical disease in the IMN. The other concern for IMNI was that it might increase cardiac mortality and compromise the survival benefit of IMNI [12–14]. Previous studies lacked comprehensive prospective assessment of cardiovascular toxicity and the follow-up was probably too short to show a significant difference in late cardiovascular toxicity. Improvements in radiation techniques, such as computed tomography (CT)-based target delineation, three-dimensional (3D) conformal radiotherapy (RT), and cardiac-sparing techniques [15], have allowed for increased target conformality and minimized heart dose, which might make IMNI safe for most patients.

According to the 2021 National Comprehensive Cancer Network (NCCN) guidelines for breast cancer, IMNI should be considered strongly when delivering regional nodal irradiation (RNI). The absence of strong evidence for or against the IMNI, has resulted in IMNI being performed selectively in real-world clinical practice; however, the optimal subgroups have not yet been identified clearly. The criteria for IMNI in different national guidelines varies, despite the guidelines being based on the same evidence. This has led to the percentages of women studied who might be considered for IMNI in different countries varying substantially, from 13 to 59% [16].

The value of IMNI in the era of modern systemic treatment and radiation techniques is still unclear; therefore, the present study aims to investigate whether IMNI improves disease-free survival (DFS) in high-risk breast cancer with pathological positive axillary nodes (pN+) after mastectomy. In addition, radiation-induced toxicity, especially cardiovascular toxicity, will be evaluated prospectively. This study will provide high-level evidence for elective IMNI for patients with breast cancer. This article was written according to the Standard Protocol Items: Recommendations for Interventional Trials (SPIRIT) guidelines, and the SPIRIT checklist is provided in supplementary material 1.

## Methods/design

### Study design

This is an open label, randomized, multi-center phase III trial for which 1800 participants will be randomized (1:1) to an IMNI group (IMNI) or a no IMN irradiation group (no IMNI).

**Fig. 1:**
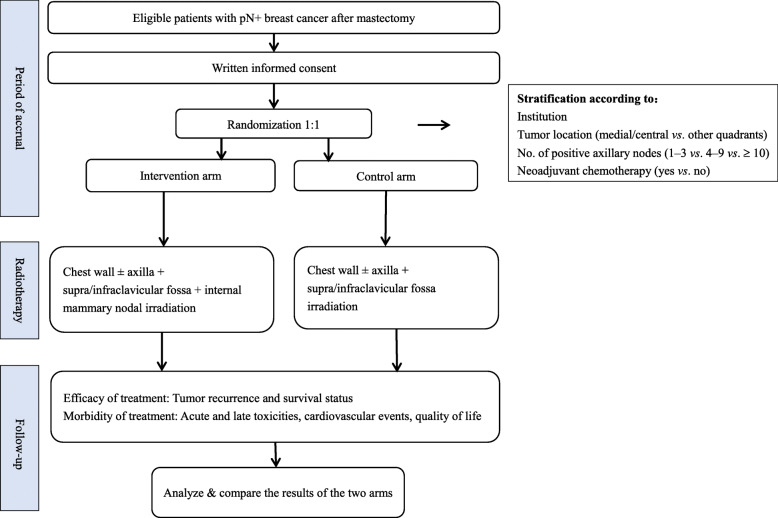
Flow chart of the POTENTIAL trial

A flow chart giving an overview of the study design is shown in Fig. 1.

### Primary endpoint

DFS with events defined as ipsilateral chest wall, axillary, supraclavicular, or internal mammary nodal recurrence, distant metastasis, contralateral invasive breast cancer, or death from any cause.

### Secondary endpoints

The effect of IMNI on overall survival (OS), IMN recurrence, locoregional recurrence (LRR), and distant metastasis (DM); the incidence of contralateral non-invasive breast cancer or other malignant tumors; the incidence of major cardiovascular toxicity or other adverse events; and Quality of life (QoL) will be assessed.

### Inclusion criteria

1. Female patients aged between 18 and 70 years old
2. Eastern Cooperative Oncology Group (ECOG) scores of 0, 1, or 2
3. Histologically confirmed invasive breast cancer
4. Patients who underwent total mastectomy and axillary dissection (10 or more axillary lymph nodes dissected) with negative margins
5. Patients who had ≥4 positive axillary lymph nodes; or 1–3 positive axillary lymph nodes and T3–4; or 1–3 positive axillary lymph nodes and T1–2, and score ≥ 3 based on the following high-risk factors: age ≤ 40 years (score 1), primary tumor involving the inner quadrant (score 1), 2–3 positive axillary lymph nodes (score 1), positive lymphovascular invasion (score 1), re-staged based on the eighth cancer staging system (IB-IIA score 1, IIB-IIIA score 2); or ypN+ (positive for lymph node metastases) after neoadjuvant chemotherapy
6. No supraclavicular or internal mammary nodal metastases based on images before and after systemic therapy
7. No distant metastases
8. Could tolerate radiotherapy
9. Treated with chemotherapy (anthracycline and/or taxane-based combined chemotherapy, ≥ six cycles)
10. Anticipated to receive endocrine therapy for 5 years if indicated
11. Anticipated to receive anti-HER2 (human epidermal growth factor receptor 2) therapy for 1 year if indicated
12. Left ventricular ejection fraction (LVEF) ≥ 50% based on an echocardiogram
13. Willing to be followed-up
14. Provided written informed consent

### Exclusion criteria

1. Simultaneous bilateral breast cancer
2. Received sentinel lymph node biopsy alone, without axillary dissection
3. Received internal mammary node dissection or sentinel node biopsy
4. No imaging assessment of the internal mammary node before systemic therapy
5. Received one-stage breast reconstruction
6. Severe cardiac insufficiency; myocardial infarction or uncorrected unstable arrhythmia or uncorrected unstable angina in the last 3 months; pericardial disease
7. Had a history of chest wall or supraclavicular radiotherapy
8. Had simultaneous or previous secondary malignancies, except for non-malignant melanoma skin cancer, papillary thyroid / follicular carcinoma, cervical carcinoma in situ, contralateral non-invasive breast cancer

### Randomization

An eligibility checklist must be completed and patient consent obtained before randomization. Eligible patients will be allocated randomly on a 1:1 basis between IMNI and no IMNI, using a computer-generated randomization scheme with a block size of four. Randomization will be stratified by institution, tumor location (medial/central or other quadrants), axillary lymph nodal burden (1–3, 4–9, ≥10 positive nodes), and neoadjuvant therapy (yes, no).

### Pre-treatment evaluation (baseline)

1. **History and physical examination**: ECOG status, height and weight, and assessment of breast cancer-related lymphedema and shoulder dysfunction
2. **Blood examination:** blood cell count; serum lipids; myocardial enzymes; thyroid hormone
3. **Image examinations:** mammography, breast ultrasonography or breast magnetic resonance imaging (MRI); contrast-enhanced CT of the neck and thorax; regional nodal ultrasonography; ultrasonography, contrast-enhanced CT or MRI of liver; bone scan or positron emission tomography (PET)/CT (recommended for patients with pN3 disease)
4. **Baseline documentation:** QoL; cardiovascular disease risk factors including being a smoker, obesity (body mass index (BMI) > 28 kg/m^2^); hypotension; diabetes; hyperlipidemia; family history of early onset cardiovascular disease (man < 55 years, woman < 65 years)
5. **Cardiac examination:** Twelve-lead electrocardiogram; echocardiography; coronary CT angiography (if the examination is accessible)

### Radiotherapy

Radiotherapy will be administered within 8 weeks after the mastectomy or the end of adjuvant chemotherapy.

In the experimental group, patients will receive irradiation to the chest wall + supraclavicular/infraclavicular fossa + IMN. Patients in the control group will receive irradiation to the chest wall + supraclavicular/infraclavicular fossa. In both groups, we recommend that the clinical target volume (CTV) of supraclavicular/infraclavicular fossa should encompass part of axilla level II on the same slices. Irradiation of level I axilla is at the discretion of the attending physician, considering the risk of axillary nodal recurrence and treatment-related lymphoedema. The delineation of the CTV of the chest wall, supra/infraclavicular fossa, and IMN (CTVcw, CTVsc, CTVim) is recommended in Table 1. The planning target volume (PTV) will be expanded from the CTV with 0.5 cm margin in all directions, but limited to 0.5 cm beneath the skin surface for PTVsc, PTVim and PTVcw2 (without bolus), and limited to skin surface for PTVcw1 (with bolus).

**Table 1:** Suggested CTV delineation

CTV	Cranial	Caudal	Medial	Lateral	Ventral	Dorsal
Chest wall	Caudal edge of the sterno-clavicular joint	Caudal edge of the contralateral breast	Within 1 cm to the midline of the body (guided by palpable/visible signs of free skin flap or the contralateral breast)	Mid-axillary line (guided by palpable/visible signs of free skin flap or the contralateral breast)	Skin surface	Anterior edge of the pectoralis minor muscle; includes the pectoralis major muscle and the interpectoral space
Supraclavicular fossa	Caudal edge of the cricoid cartilage	5 mm below the subclavian vein	Medial edge of the internal jugular vein, excluding the thyroid and the common carotid artery	Medial edge of the trapezius muscle and the pectoralis minor muscle	Posterior surface of the sternocleido mastoid muscle and platysma muscle	Ventral surface of the scalene muscle bundle
Infraclavicular fossa (Axilla level III)	Pectoralis minor muscle inserts on the coracoid process	5 mm caudal to the axillary vein cross medial edge of the pectoralis minor muscle	Medial edge of the collar bone and ribs; lateral edge of the junction of the subclavian and internal jugular veins	Medial edge of the pectoralis minor muscle	Posterior surface of the pectoralis major muscle	Dorsal to the subclavian vein and axillary vein (anterior edge of the ribs)
Axilla level II	Cranial edge of the pectoralis minor muscle	Caudal edge of the pectoralis minor muscle	Medial edge of the pectoralis minor muscle	Lateral edge of the pectoralis minor muscle	Posterior surface of the pectoralis major muscle	5 mm dorsal of the axillary vein, or the ventral edge of the ribs and the intercostal muscle
Axilla level I	Axillary vein cross lateral edge of the pectoralis minor muscle	To the level of rib 4–5	Lateral edge of the pectoralis minor muscle	Medial edge of the latissimus dorsi muscle	5 mm anterior the plane defined by the anterior surface of the pectoralis major muscle and the latissimus dorsi muscle	Anterior surface of the subscapularis muscle
IMN region	5 mm caudal to the subclavian vein, thus connecting to the caudal border of the infraclavicular fossa	Cranial edge of the fourth rib	5 mm medial to IM vessels	5 mm lateral to IM vessels	5 mm anterior to IM vessels, excluding the chest wall	5 mm posterior to IM vessels, excluding the lung

Abbreviations: CTV, clinical target volume; IMN, internal mammary nodal

The prescribed dose is either 50 Gy in 25 fractions over 5 weeks at 2 Gy per daily fraction, or 43.5 Gy in 15 fractions over 3 weeks at 2.9 Gy per daily fraction. CT-based 3D treatment plans are required, including 3D conformal RT, intensity-modulated RT (IMRT), or volumetric modulated arc therapy (VMAT). It is required that 95% of the PTV receives 100% of the prescribed dose, the maximum dose (Dmax) of the PTV < 120% of the prescribed dose, and the PTV receiving ≥110% of the prescribed dose is specified to be < 25%. Electron fields are optional for irradiation of the chest wall and the IMN. When an electron field is used, it is required that 90% of the CTVim or CTVcw receive 90% of the prescribed dose. Chest wall bolus should be used for at least 40–60% of the course of RT. Recommended dose constraints for organs at risk (OARs) are shown in Table 2. Position verification before delivering RT will be performed on a linear accelerator (LINAC) at least once per week with an electronic portal imaging device (EPID) or cone beam computed tomography (CBCT).

**Table 2:** Suggested dose constraints of organs at risk

Organs at risk	43.5 Gy in 15 fractions	50 Gy in 25 fractions
Heart (left-sided breast cancer)	Dmean < 8 Gy	Dmean < 10 Gy
	V5 < 45%	V5 < 50%
Heart (right-sided breast cancer)	Dmean < 5 Gy	Dmean < 6 Gy
	V5 < 30%	V5 < 35%
Left anterior descending coronary artery	V40 < 2 0%	V40 < 20%
Right coronary artery	V40 < 20%	V40 < 20%
Ipsilateral lung	Dmean < 15 Gy	Dmean < 15 Gy
	V20 < 30%	V20 < 30%
	V5 < 55%	V5 < 55%
Contralateral lung	V5 < 20%	V5 < 20%
Contralateral breast	Dmean < 5 Gy	Dmean < 5 Gy
Spinal Cord PRV	Dmax < 30 Gy	Dmax < 40 Gy
Esophagus/Ipsilateral Brachial plexus	Dmax < 48 Gy	Dmax < 55 Gy
Ipsilateral shoulder joint	V30 < 30%	V30 < 30%
Thyroid gland	Dmean < 28 Gy	Dmean < 3 0 Gy
Liver (right-sided breast cancer)	V5 < 25%	V5 < 25%
Stomach (left-sided breast cancer)	V 5 < 25%	V5 < 25%
Liver (left-sided breast cancer)	V5 < 10%	V5 < 10%
Stomach (right-sided breast cancer)	V5 < 10%	V5 < 10%

Abbreviations: Dmean, mean dose; Vx, percent volume of the structure receiving x Gy; Dmax, maximal dose

### Follow-up

The patients will be followed up for at least 10 years after treatment. During follow-up visits, the oncological and toxilogical outcomes will be evaluated during RT; at 1, 2 weeks and 3, 6 months after RT; every 6 months for the first 5 years, and then annually. All recurrences should be confirmed by histology or cytology if possible. Acute radiation toxicity will be graded according to the National Cancer Institute’s Common Terminology Criteria for Adverse Events version 3.0 (CTCAE 3.0). Late radiation toxicity will be graded according to the Radiation Therapy Oncology Group (RTOG)/the European Organization for Research and Treatment of Cancer (EORTC) late radiation morbidity scale, and QoL assessment will be performed according to patient-reported questionnaires (EORTC QLQ-C30 and QLQ-BR23). Table 3 shows the follow-up schedule.

**Table 3:** Follow-up workflow

	Pre-RT	During RT		6 months after RT				6 months to 5 years after RT	5–10 years after RT
	baseline	weekly	end	1 week	2 weeks	3 months	6 months	every 6 months	annually
History and physical exam	X	X	X	X	X	X	X	X	X
Complete blood cell count	X	X	X	X	X	X	X	X	X
Thyroid function (Blood analysis)	X					X	X	X	X
†Myocardial enzyme spectrum	X					X	X	X	X
Chest CT	X					X	X	X	X
Regional nodal ultrasonography	X					X	X	X	X
Ultrasonography/CT/MRI for liver	X					X	X	X	X
Twelve-lead electrocardiogram	X					X	X	X	X
Echocardiography	X					X	X	X	X
†‡Coronary CT angiography	X							(1) 3 years	6, 10 years
Quality of life	X		X			X	X	X	

† Applicable to patients for whom the examination is accessible

‡ The frequency of coronary CT angiography examination is dependent on patient age and the baseline status of coronary artery stenosis. For patients younger than 65 years and with less than 50% stenosis in left anterior descending coronary artery (LAD), left circumflex artery (LCX) and right coronary artery (RCA), the coronary CT angiography examination is scheduled at 3, 6, and 10 years after RT. If the stenosis severity in one of the three main coronary arteries exceeds 50% and/or the patient is older than 65 years, the coronary CT angiography examination is scheduled at 1, 3, 6, and 10 years after RT. If the stenosis severity in one of the three main coronary arteries exceeds 75%, revascularization should be performed and the coronary CT angiography examination is scheduled at 3, 6, and 10 years after RT

### Statistical analysis & sample size calculation

The primary end point is DFS. We hypothesize that IMNI group has superior 5-year DFS to the no IMNI group. Based on previous studies, the 5-year DFS rate was 70–74% for patients with node-positive breast cancer who did not receive IMNI [11, 17]. To detect a 6% difference in the 5-year DFS (78% in the IMNI group and 72% in the no IMNI group; equivalent to a Hazard Ratio [HR] of 0.76), with a power of 80%, and a one-sided alpha value of 2.5%, a total of 414 events are required. With an anticipation of recruitment during a 5-year period, the accrual target to randomize is 1800 patients.

Patients will be analyzed according to the intention to treat principle. Two interim analyses for efficacy and futility are planned to occur when 207 DFS events (50% information) and 311 DFS events (75% information) are observed. The critical values for the interim analyses will be determined using the Lan–DeMets implementation of the O’Brien–Fleming boundary for interim efficacy analysis and a linear 20% inefficacy boundary for futility analysis [18]. The principal investigator and the protocol committee will perform the analysis. A Data Monitoring Committee (DMC) will be set up to oversee the trial and to decide whether the trial should be stopped.

### Radiotherapy quality assurance

According to the guidelines for quality assurance (QA) protocol of individual trials laid down by the US National Cancer Institute Work Group on Radiotherapy Quality Assurance [19], the POTENTIAL trial will formulate a tiered RT QA protocol requirement, including general credentialing, trial specific credentialing, and individual case review. Before opening inclusion, all participating centers will provide the qualified documents of the QA process of the CT simulator, LINAC, and the RT planning system for general credentialing. To ensure quality and uniformity between centers, delineation and treatment planning guidelines are described in detail in the protocol. All participating centers will perform a dry run, focusing on target delineation, RT planning generation, and planning QA on phantoms, to assess compliance with the protocols. During inclusion, central individual case review on the target delineation, RT planning, and treatment position verification data will be performed prospectively for the first 10 patients included at each participating center and an additional 10 patients if major deviation is found in 2 out of the first 10 patients, which then will be performed randomly for 5% of all included patients thereafter.

### Ethics

The study protocol has been approved by the ethics committee of the Cancer Hospital, Chinese Academy of Medical Sciences (19/317–2101). The study is registered in ClinicalTrails.gov (NCT04320979).

### Trial status

Recruitment started in May 2020 and is currently ongoing.

## Discussion

In this report, we present the design of the POTENTIAL trial, which aims to investigate whether IMNI can improve DFS in high-risk breast cancer with pN+ after mastectomy. We also intend to determine which subgroups of patients might derive an OS benefit from IMNI. The results from studies designed to evaluate the effect of IMNI itself were conflicting. Some retrospective studies showed that delivering IMNI could improve DFS significantly compared with not delivering IMNI [20–25], whereas other studies demonstrated no benefit of IMNI on DFS [26–28]. Only a few studies observed that for some subgroups of patients with high-risk factors, such as ypN1 (1–3 positive nodes among all axillary nodes harvested) disease, node-positive disease, inner/central tumor, clinical stage II–III breast cancer before neoadjuvant systemic therapy, there is a trend toward improved OS with IMNI [20, 23–26]. The DBCG-IMN prospective study suggested that IMNI improved 8-year OS from 72.2 to 75.9% (HR = 0.82, *p* = 0.005), and greater OS benefit was seen in subgroups of patients with ≥4 positive nodes, or with 1–3 positive nodes and medial tumors [11]. By contrast, the French randomized study was the only one to evaluate postmastectomy IMNI itself and showed no OS benefit from IMNI in the entire cohort or any subgroup of patients [10]. This failure to demonstrate a survival benefit for IMNI in the French study could be explained as follows. Firstly, low-risk patients, such as patients with pN0–1 disease, accounted for a major proportion of the cohort, thus the overestimation of the risk of IMN involvement probably decreased the power of the study. Another retrospective study included patients after breast-conserving surgery, most of whom had pN0 disease or did not undergo axillary lymph node dissection, and also showed that no benefit could be attributed to IMNI [28]. Secondly, the study intended to detect a 10% difference in OS between the IMNI group and the no IMNI group at 10 years, which might have been too optimistic. Moreover, quality control of RT was not planned and deviations from the protocol might have compromised the effect of IMNI; RT was performed in the 2-dimensional era, thus the IMN area could have been partially irradiated in some patients in the IMNI group, and protection of the heart could not be performed routinely. Lastly, the systemic therapy used was weak compared with current practice.

In our study, we will evaluate IMNI in high-risk patients who were treated with modern systemic therapy and 3D RT techniques. The risk of IMN involvement is associated significantly with the axillary lymph node burden. The incidence of IMN involvement was 4.4, 18.8, and 58.2% for patients with 0, 1–3 and ≥ 4 positive axillary lymph nodes [5]. Considering that most of the patients with pN0 disease do not have indications to receive PMRT, we will only include patients with pN+ disease. With regard to patients with T1–2 disease and 1–3 positive axillary lymph nodes, we will include the high-risk subset defined according to several risk factors based on our own retrospective data [29]. We choose DFS, rather than OS, as the primary endpoint, because the results of OS require longer follow-up and a larger patient population. Patients tend to survive longer after disease relapse because of the rapid development of effective salvage systemic therapy. Results from clinical trials will forever lag behind developments in clinical practice [11], which might not be applicable to patients treated in the future.

The value of IMNI for OS might be compromised by radiation-induced cardiac mortality, despite the DFS benefit. Cardiovascular toxicity is an important secondary endpoint in this study, which is assessed regularly by cardiologists to avoid an incomplete reporting of cardiac events [30]. IMNI will increase the heart dose [31] and has the potential to increase the risk of cardiac mortality, because there is a significant dose-effect relationship between acute coronary events or heart disease mortality and the mean heart dose [30]. In the protocol, the dose constraints to the heart and coronary arteries are required to be respected. Oncocardiology is an emerging field in cardiovascular and cancer healthcare [32]. We have developed an oncocardiology service for patients with breast cancer through close cooperation between cardiologists and oncologists. In this study, identification of baseline cardiac risk factors and early detection of radiation-induced cardiac damage, followed by adoption of preventive or therapeutic interventional strategies, might present the potential to reduce cardiac mortality.

Radiotherapy QA might affect clinical trial accrual, cost, outcomes, and generalizability, and is integral to this trial design. QA of non-CT-based IMNI in the DBCG study, using modern techniques to derive estimates of the doses to the IMN and OARs, showed that RT techniques utilizing electrons for IMNI had a high degree of IMN dose coverage with little variation in the patient population. However, in techniques using tangential fields for IMNI, estimates on IMN dose coverage were more uncertain, and some patients are likely to have received lower than the intended IMN dose [33]. A dummy run of the QA program in the Korean Radiation Oncology Group 08–06 Study, which is a phase 3 randomized trial designed to investigate the role of IMNI in breast cancer, revealed that various 3D RT techniques were adopted among participating institutions, and an average of 59.0% of the prescribed dose was delivered to the IMN in the no IMNI group, which was higher than expected because of the close proximity of the IMN to the medial margin of the chest [34]. According to our RT QA protocol, we will assess IMN delineation and evaluate the dosimetric parameters of the IMN and OARs. Careful data analysis and feedback will reduce inter-institutional heterogeneities and deviations from the protocol, which might help to provide reliable trial results.

## Conclusion

In conclusion, the POTENTIAL study is a pragmatic randomized trial that compares survival and toxicity outcomes of delivering IMNI with not delivering postmastectomy IMNI in patients with node positive breast cancer. We anticipate that the results of this trial will provide high-level type I evidence on IMNI that could improve the decision-making process.

## Supplementary Information

**Additional file 1.**

## Data Availability

Data sharing is not applicable to this article as the current study is still open for inclusion of patients.

## Abbreviations

RT: radiotherapy
IMN: internal mammary node
IMNI: internal mammary node irradiation
3D: three-dimensional
DFS: disease-free survival
PMRT: postmastectomy radiation therapy
OS: overall survival
DBCG: Danish Breast Cancer Cooperative Group
CT: computed tomography
NCCN: National Comprehensive Cancer Network
RNI: regional nodal irradiation
LRR: locoregional recurrence
DM: distant metastasis
QoL: quality of life
ECOG: Eastern Cooperative Oncology Group
LVEF: left ventricular ejection fraction
PET: positron emission tomography
CTV: clinical target volume
PTV: planning target volume
IMRT: intensity-modulated RT
VMAT: volumetric modulated arc therapy
Dmax: maximum dose
OARs: organs at risk
EPID: electronic portal imaging device
CBCT: cone beam computed tomography
Dmean: mean dose
Vx: percent volume of the structure receiving x Gy
CTCAE 3.0: National Cancer Institute’s Common Terminology Criteria for Adverse Events version 3.0
RTOG: Radiation Therapy Oncology Group
EORTC: European Organization for Research and Treatment of Cancer
QA: quality assurance
